# Focused Screening and Treatment (FSAT): A PCR-Based Strategy to Detect Malaria Parasite Carriers and Contain Drug Resistant *P. falciparum*, Pailin, Cambodia

**DOI:** 10.1371/journal.pone.0045797

**Published:** 2012-10-01

**Authors:** Stefan Hoyer, Sokomar Nguon, Saorin Kim, Najibullah Habib, Nimol Khim, Sarorn Sum, Eva-Maria Christophel, Steven Bjorge, Andrew Thomson, Sim Kheng, Nguon Chea, Sovann Yok, Samphornarann Top, Seyha Ros, Uth Sophal, Michelle M. Thompson, Steve Mellor, Frédéric Ariey, Benoit Witkowski, Chhiang Yeang, Shunmay Yeung, Socheat Duong, Robert D. Newman, Didier Menard

**Affiliations:** 1 World Health Organization, Global Malaria Programme, Geneva, Switzerland; 2 National Center for Parasitology, Entomology, and Malaria Control, Phnom Penh, Cambodia; 3 Malaria Molecular Epidemiology Unit, Institut Pasteur in Cambodia, Phnom Penh, Cambodia; 4 World Health Organization, Phnom Penh, Cambodia; 5 World Health Organization, Regional Office for the Western Pacific, Manilla, Philippines; 6 Malaria Consortium, Phnom Penh, Cambodia; 7 Unité d'Immunologie Moléculaire des Parasites, Institut Pasteur, Paris, France; 8 Malaria Centre, London School of Hygiene and Tropical Medicine, London, United Kingdom; Johns Hopkins University, United States of America

## Abstract

Recent studies have shown that *Plasmodium falciparum* malaria parasites in Pailin province, along the border between Thailand and Cambodia, have become resistant to artemisinin derivatives. To better define the epidemiology of *P. falciparum* populations and to assess the risk of the possible spread of these parasites outside Pailin, a new epidemiological tool named “Focused Screening and Treatment” (FSAT), based on active molecular detection of asymptomatic parasite carriers was introduced in 2010. Cross-sectional malariometric surveys using PCR were carried out in 20 out of 109 villages in Pailin province. Individuals detected as *P. falciparum* carriers were treated with atovaquone-proguanil combination plus a single dose of primaquine if the patient was non-G6PD deficient. Interviews were conducted to elicit history of cross-border travel that might contribute to the spread of artemisinin-resistant parasites. After directly observed treatment, patients were followed up and re-examined on day 7 and day 28. Among 6931 individuals screened, prevalence of *P. falciparum* carriers was less than 1%, of whom 96% were asymptomatic. Only 1.6% of the individuals had a travel history or plans to go outside Cambodia, with none of those tested being positive for *P. falciparum*. Retrospective analysis, using 2010 routine surveillance data, showed significant differences in the prevalence of asymptomatic carriers discovered by FSAT between villages classified as “high risk” and “low risk” based on malaria incidence data. All positive individuals treated and followed-up until day 28 were cured. No mutant-type allele related to atovaquone resistance was found. FSAT is a potentially useful tool to detect, treat and track clusters of asymptomatic carriers of *P. falciparum* along with providing valuable epidemiological information regarding cross-border movements of potential malaria parasite carriers and parasite gene flow.

## Introduction

The border region between Cambodia and Thailand, specifically in Pailin province, has repeatedly been the epicenter of emerging resistance of *Plasmodium falciparum* to antimalarial drugs since the 1960s [1]. This started with resistance to chloroquine (CQ) first reported in 1959 [2], followed by resistance to sulfadoxine-pyrimethamine in 1980's [3] and, more recently, mefloquine [4], [5]. In response to increasing levels of drug resistance, Cambodian and Thai national malaria control programmes adapted their national treatment policies. Artemisinin-based combination therapies (ACTs) were implemented as the first-line treatment for uncomplicated falciparum malaria in Thailand in 1995 and in Cambodia in 2000. During the last decade, results from annual routine therapeutic efficacy studies conducted in Cambodia have demonstrated that the efficacy of artesunate-mefloquine and artemether-lumefantrine for the treatment of falciparum malaria was declining in several areas of the country [6]–[8]. In 2007, the World Health Organization (WHO) initiated an informal consultative workshop in Phnom Penh, Cambodia, during which available evidence regarding the emergence of *P. falciparum* AR parasites was reviewed [9]. Following this meeting, a comprehensive clinical study clearly demonstrated that parasite clearance times were uniformly delayed in patients in Pailin compared to patients at the Myanmar-Thailand border, justifying further research and action [10]. In this context, in order to prevent the possible spread of *P. falciparum* AR parasites, the “Artemisinin-Resistance Containment Project” was implemented by the governments of Cambodia and Thailand with coordination and technical support by WHO and partners with funding from the Bill & Melinda Gates Foundation. Its overall goal was to contain artemisinin-tolerant *P. falciparum* parasites by removing selection pressure and reducing and ultimately eliminating falciparum malaria. In order to achieve this goal, an ambitious programme of activities was carried out, including the provision of universal coverage with long-lasting insecticide treated nets (LLINs) and of rapid access to free, parasitologically confirmed diagnosis through rapid diagnostic test (RDT) and ACTs delivered by community-based Village Malaria Workers (VMWs).

Initially, a much wider operation referred to as “Mass Screening and Treatment” (MSAT), which aims at screening and treating the entire population of Pailin, estimated at over 70,000, within two weeks was envisaged but abandoned following two pilot studies conducted in November 2008 and May 2009. It was therefore decided to carry out to evaluate Focused Screening and Treatment (FSAT), a PCR-based strategy intended to detect *P. falciparum* asymptomatic carriers. PCR-based cross sectional screenings were focused on villages with the highest annual incidence rates for acute *P. falciparum* malaria infections reported by heath centers (HC) through malaria microscopy and by VMWs using RDT. Based on high-throughput molecular diagnosis of malaria, the present paper compiles information obtained through the FSAT pilot with a view to gaining more micro-epidemiological knowledge about the population of asymptomatic carriers which would otherwise have remained largely undetected.

## Materials and Methods

### Study sites & Participants

**Figure 1:**
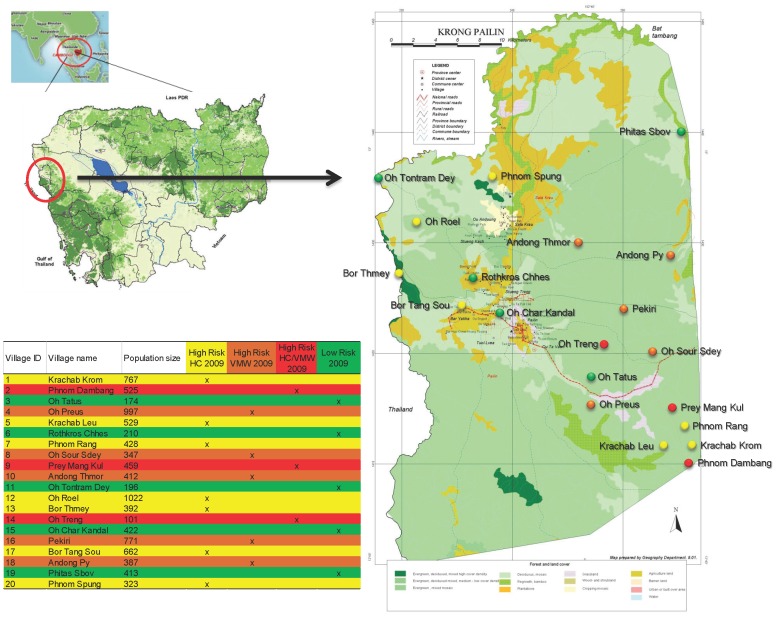
Spatial distribution of the selected villages, Pailin, Cambodia, 2010.

Based on the observation that the incidence of parasitologically confirmed malaria cases in general and that of *P. falciparum* in particular was highly clustered in Pailin, the 109 villages of Pailin were split into two groups: “high risk” villages and “low risk” villages based on available records of parasitologically confirmed symptomatic cases of *P. falciparum* malaria (2009). The “high risk” village group consisted of the 10 villages reporting the highest *P. falciparum* incidence rates at HC and of 8 villages reporting the highest number of cases through VMWs in 2009. All others were classified as “low risk” villages. As three villages of Pailin qualified for “high risk” in both categories (HC and VMW records), only 15 out of Pailin's 109 villages were identified as “high risk”, the remaining 94 were classified as “low risk”. All 15 “high risk” villages were selected, comprising among them 71% of all parasitologically confirmed cases of *P. falciparum* malaria recorded in Pailin in 2009 (623/941). The 2009 village selection threshold between “high risk” and “low risk” villages was >8 cases of *P. falciparum* with high risk villages reporting 9 to 78 (average 41.5) cases and low risk reporting 0 to 7 (average 3.4) cases throughout 2009 (CNM and Malaria Consortium, unpublished data). The “high risk” villages included seven villages (Krachab Krom, Krachab Leu, Phnom Rang, Oh Roel, Bor Thmey, Bor Tang Sou and Phnom Spung) classified as “high risk HC 2009”, five villages (Oh Preus, Oh Sour Sdey, Andong Thmor, Pekiri and Andong Py) classified as “high risk VMW 2009” and three villages (Phnom Dambang, Prey Mang Kul, Oh Treng) classified as “high risk HC/VMW 2009”. Five villages (Oh Tatus, Rothkros Chhes, Oh Tontram Dey, Oh Char Kandal, Phitas Sbov) were randomly selected among the remaining 94 of 109 villages, classified as “low risk 2009” (Figure 1).

### Field procedures: samples collection and workflow of the screening phase

FSAT interventions were conducted at a rate of 3 villages per month. The Screening Team (ST) of seven persons spent four days in each village. The ST was followed at a one-week interval by the Follow-up and Treatment Team of two persons (FTT). Both ST and FTT received local support by the active participation of the village chief in identifying the individuals participating as well as the VMW assisting the direct observation of treatment of those found positive by RDT or PCR. The ST proceeded to the next village and so on until it had screened three villages, before taking one week's rest. During the 4^th^ week, the FTT completed the third village and rested during week 5. In the week prior to commencement of screening, the ST members visited the village and met the village chief and the VMW. They conducted a detailed joint planning discussion based on the village's logistic and geographical organization (examining the village family register, gaining an idea of how many individuals were likely to turn up at screening, and following recommendations of the village chief on the best locations to set up the screening). Procedures to screen individuals were divided among 4 dedicated stations:

#### Registration and Informed Consent

Two ST members, assisted by the village chief, located each villager in the existing village register. The villager's name was manually transcribed into the FSAT screening register with a unique ID code (village number+family number+letter designating position in the family+screening sequence number). Absent family members were also recorded. In order to assess the risk of spread of drug resistance, the individuals were questioned regarding travel over the past year and classified as “risk class A” (travel to or from Myanmar), “risk class B” (travel to or from Thailand), “risk class C” (travel within Pailin Province only) and “risk class D” (travel elsewhere within Cambodia). The screening process was explained to each villager, prior to signing or thumb-printing of the consent form.

#### Temperature Check

Axillary temperature was recorded for each participant and the result transmitted immediately back to the registration and informed consent station. Individuals with temperature ≥37.2°C received an additional mark on their arm, indicating that they would require a malaria rapid diagnostic test (RDT).

#### Laboratory Station

Any individuals detected as being sub-febrile or febrile were tested for malaria by using a RDT (CareStart©, Access Bio Inc®, Somerset, NJ, USA), detecting falciparum malaria (HRP-2) and non-falciparum malaria (lactate dehydrogenase). If the result was positive, a mark was made on the villager's forearm, identifying him/her for the malaria treatment station. For all participants in the survey, finger prick blood samples were collected and used to perform thick and thin films and double blood spots (one blood spot in 96 well-format PCR plate and one classical blood spot as backup). For quality control, in each 96 well-format PCR plate, one field positive and one negative blood spot controls was included by the ST and blinded to laboratory staff at the Pasteur Institute in Cambodia (IPC) in Phnom Penh, which carried out the PCR tests.

#### Treatment Station

At the treatment station, RDT positive cases were treated for *P. falciparum* with atovaquone-proguanil (AP, Malarone®, GlaxoSmithKline®) according to manufacturer's instruction or with CQ for non-*P. falciparum* infections (chloroquine phosphate Tablets®, Medopharm®), according to the national treatment guideline. Prior to treatment, pregnant women were detected through questioning and a pregnancy rapid diagnostic test, if necessary. Pregnant women received quinine (10 mg/kg, eight-hourly×7 days). As soon as the patient received the first dose of treatment, a detailed follow-up interview was conducted. RDT negative cases received multivitamins and anti-helminthic treatment, and if symptomatic with fever and other clinical signs of bacterial infection, co-trimoxazole. At the completion of screening in each village, slides and dried blood spot samples were transported within 24 hours to the IPC laboratory in Phnom Penh.

### Field procedures: follow-up of PCR-positive cases

Follow-up was triggered when the list of PCR positive cases including species identification, identification of Y268N mutation in cytochrome *b* gene and G6PD status was transmitted to the FTT from IPC by SMS. PCR-confirmed cases of *P. falciparum* infections were treated with AP in absence of mutation at codon 268 in cytochrome *b* gene. Direct observed treatment at dosages following manufacturer recommendation was carried out over three days. Malaria cases were followed up daily for the first three days and on day 7 and day 28. Primaquine (PQ) was used according to G6PD status. Individuals with normal G6PD levels and infected with *P. falciparum* received a single dose of PQ (0.75 mg/kg, Cipla Ltd®, India) under supervision on day 2, while normal G6PD infected by *P. vivax* received CQ over three days according to WHO guidelines and PQ for 14 days (0. 50 mg/kg). Any patients presenting with severe symptoms were given emergency support and referred to the Pailin referral hospital for further assessment and management. Blood was obtained by finger prick on all follow-up days and on any unscheduled day to use for analysis of thick and thin blood smears and for storage on filter paper.

### Treatment outcomes

Treatment outcomes were classified as treatment failure if the patient presented with recurrent asexual parasitemia at day 7 or day 28 and treatment success if both day 7 and day 28 asexual parasitemia were negative. According to the national treatment policy, quinine (10 mg/kg, eight-hourly×7 days) plus tetracycline (8.3 mg/kg, eight-hourly×7 days) was used at Pailin referral hospital to treat *P. falciparum* recrudescent cases. DHA-PIP (Duo-Cotexin®, Zhejiang Holley Nanhu Pharmaceutical Co. Ltd®) was used for non-*P. falciparum* recrudescent cases.

### Laboratory procedures

#### Microscopy examination

Microscopy blood slides were stored in order to cross-check all RDT and PCR positive samples. Blood smears were stained with 3% Giemsa for 30 min at room temperature. Asexual- and sexual-stage parasite counts were performed by two experienced microscopists. In the case of a discordant result, a third reading was done. Parasite densities were determined from thick blood smears by counting the number of asexual parasites per 200 WBCs (or per 500 WNCs, if the count was less than 10 parasites/200 WBCs), assuming a WBC count of 8,000/µl. A smear was considered negative if no parasites were seen after review of 100 high-power fields.

#### DNA extraction

DNA from blood spot collected in 96-well PCR plate format containing patient screening samples and field positive and negative controls were extracted with Instagene Matrix resin® (Bio-Rad®, France), according to the protocol adapted from the suppliers recommendations and previously described by Steenkeste et *al.* [11].

#### Nested PCR detection

Detection of *Plasmodium* was carried out using primers targeting the *Plasmodium* cytochrome b gene as described by Steenkeste et *al.* [11]. For positive samples, RFLP on PCR products using AluI enzyme was performed for determining *Plasmodium* species. Briefly, 5 µl of PCR products adjusted to 500 ng/µl were mixed with 0.2 µl of AluI and 2.5 µl of buffer A according to the manufacturer's instructions (New England Biolabs®, France), incubated for 4 hours at 37°C and inactivated at 65°C for 15 minutes. Bands were detected by standard 2% agarose gel electrophoresis and ethidium bromide staining. *Plasmodium* species identification was assessed according to the number and the size of the bands: *P. falciparum* (2 bands, 640 and 159 bp), *P. vivax* (5 bands, 270, 249, 187, 169 and 111 bp), *P. ovale* (2 bands, 584 and 224 bp) and *P. malariae* (3 bands, 381, 249 and 187 bp).

#### Detection of atovaquone resistance (codon 268)

All *P. falciparum* positive samples were screened for the Y268 mutations in cytochrome *b* gene that have been related to atovaquone resistance. PCR products from the outer PCR used to detect malaria parasites (1385 pb) were used as template for two second round PCRs. For both nested PCR (NsiI and SspI), amplification took place in the following reaction mixture: 2.5 µl of 10× buffer, 1.5 mM MgCl2, 0.2 mM each deoxynucleoside triphosphate, 0.5 µM each primer (forward primer NsiI, 5′-ggtttacttggaacagtttttaacaatg-3′, reverse primer NsiI, 5′-ggtttacttggaacagtttttaacaatg-3′ and forward primer SspI, 5′-acagaataatctctagcacc-3′, reverse primer NsiI, 5′-acctgaatggtactttctacaatat-3′), 2 U of FirePol Taq polymerase (Solis Biodyne®, Estonia), and 2 µl of PCR products. Nested PCRs were performed under the following conditions: heating at 94°C for 5 min, followed by 30 cycles of heating at 94°C for 30 s, 45°C (nested PCR NsiI) or 55°C (nested PCR SspI) for 90 s, and 72°C for 2 min, and a final extension period at 72°C for 10 min. Five µl of nested PCR products adjusted to 500 ng/µl were respectively mixed with 0.2 µl of NsiI and 2.5 µl of buffer 3 (nested PCR NsiI) and with 0.2 µl of SspI and 2.5 µl of buffer 2 (nested PCR SspI) according to the manufacturer's instructions (New England Biolabs®, France), incubated for 4 hours at 37°C and inactivated at 65°C (SspI) or 80°C (NsiI) for 20 minutes. Bands were detected by standard 2% agarose gel electrophoresis and ethidium bromide staining. Polymorphism at codon 268 was assessed according to the number and the size of the bands: Y268Y (tat) - NsiI RFLP (2 bands, 359 and 25 bp) and SspI (2 bands, 151 and 23 bp); Y268N (aat) - NsiI RFLP (2 bands, 359 and 25 bp) and SspI (1 band, 174 bp); Y268C (tgt) or Y268S (tct) - NsiI RFLP (1 band, 384 bp) and SspI (2 bands, 151 and 23 bp).

#### Sequencing reactions

*Plasmodium* species identification and mutations at codon 268 were secondly confirmed by sequencing. Briefly, nested PCR products were purified by 96-well plate filtration (Millipore®, France) using a polyacrylamide gel (Bio-Gel P-100®, Bio-Rad®, France). Sequencing reactions were carried out with the ABI Prism BigDye Terminator cycle sequencing ready reaction kit run on a 3730 XL genetic analyzer (Applied Biosystems®, France). Electrophoregrams were visualized and analyzed with Seqscape software v2. 0 (Applied Biosystems®, France). Amino acid sequences were compared with the following sequences: GenBank accession no. NC002375 for *P. falciparum*, AY598138 for *P. vivax*, NC007232 for *P. knowlesi*, AF069624 for *P. malariae* and AB182496 for *P. ovale*, using BioEdit Sequence Alignment Editor software [12].

#### Detection of G6PD deficiency

To determine which individuals should receive primaquine treatment, all individuals who tested PCR positive were screened for G6PD deficiency from backup blood spots according to the protocol developed by Kuwahata et *al* [13]. As this method turned out to be unreliable in identifying truly G6PD deficient individuals, by systematic over-reporting, only those found to be surely not G6PD deficient received primaquine, while an unknown number of false positives in the test had to be excluded from receiving the additional gametocidal treatment.

#### *P. falciparum* genotyping

Molecular genotyping techniques were used to distinguish recrudescence from new infection for all patients who failed therapy after day 7. Filter paper blood samples collected on the day of enrolment and on the day of failure were analyzed for polymorphisms in the genes for merozoite surface protein 1 (*msp-1*), merozoite surface protein 2 (*msp-2*) and glutamate rich-protein (*glurp*) using nested-PCR as described elsewhere [14]. In accordance with WHO recommendations [15], treatment failure was labeled recrudescence if all *msp1*, *msp2* and *glurp* alleles present at the time of failure had been present at the time of treatment initiation. In all other cases, the failure was considered a new infection.

#### *P. falciparum* bar-coding

Bar-coding assays were performed as previously described by Daniels et *al.* [16] with minor modifications. Polymorphisms in eight SNPs were assessed according to Daniels et *al.*'s assay numbering (assay number 3, 4, 7, 8, 9, 19, 20, 24) by nested PCR approach (Table S1). Second PCR amplification was immediately followed by HRM ramping from 65°C to 85°C with fluorescence data acquisition set at 0.2°C increments. HRM analysis was performed by software supplied by Biorad® (CFX Manager 2. 1®, Bio-Rad®, France), assigning automatically the two possible nucleotides for each sample. In each run, samples were analyzed in duplicate with 3D7 and Dd2 genomic DNA controls and no template control.

### Statistical analysis

Data were entered and verified using Microsoft Excel software® and analyzed using XLSTAT for Windows XP® (Addinsoft®, France) and MedCalc software (version 9. 1. 0. 1, Belgium). The Mann-Whitney U test or Kruskal-Wallis method were used for non-parametric comparisons, and Student's t test or one-way analysis of variance for parametric comparisons. For categorical variables, Chi-squared or Fisher's exact tests were used to assess significant differences in proportions. The efficacy outcomes were 28-day clinical and parasitological failure risks adjusted by genotyping, in accordance with the 2009 WHO protocol [17]. Hypothesis testing was made using risk differences, exact 95% confidence intervals, and *P*-values. A *P*-value (two-tailed) of less than 0.05 was considered statistically significant.

Genetic diversity was assessed by Nei's unbiased expected heterozygosity (*He*) from haploid data and calculated as *He* = [n/(n−1)][1−pi] (n = the number of isolates sampled; pi = the frequency of the ith allele [18]. Population genetic differentiation was measured using Wright's F statistics [19]. Population genetic parameters were computed with FSTAT software, v2. 9. 4 [20].

### Ethical clearance

The study protocol was reviewed and approved by the Cambodian National Ethics Committee on Health Research (NECHR) and by the Ethics Board of the WHO Western Pacific Region office (WPRO). An informed written consent was provided by the parents/guardians of all patients before testing.

## Results

### Population screening

The FSAT study was conducted from May to October 2010. A total of 6,931 individuals were screened in the 20 selected villages. The coverage achieved was 72.7% when counting all persons present at the village at the moment of FSAT and their relatives who were known but absent (6,931/9,537). The mean age of the screened population was 23.7 years (SD, ±17.1 years), ranging from 1 week to 89 years. The mean gender ratio (male/female) was 1.04, ranging from 1.32 (in Oh Tatus and Oh Trengleu villages) to 0.88 (Bor Thmey). Risk classification according to the movement of individuals showed that none of individuals declared that they had come from or were be likely to go to Myanmar (“risk class A”); 139 (1.6%) were identified to have a travel history to or from Thailand and were classified as belonging to “risk class B”; 8550 (94. 6%) declared that they had stayed or were likely to stay within Pailin or local transmission areas within Cambodia (“risk class C”); and 341 (3.8%) declared that they had come from or were likely to proceed to non-malaria transmission areas within Cambodia (“risk class D”). Three individuals (1 in Phnom Dambang and 2 in Oh Preus) were said to work as maids in Malaysia and were initially falsely classified as “risk class A”. However, given that they were most likely to work in an urban area they were considered not be posing a risk to the spread of malaria parasites from Pailin, they were removed from “risk class A” and remain unclassified. No significant differences were observed between villages with regard to risk classes. In the 20 villages, 621 individuals (8.9%) were febrile or sub-febrile with axillary temperature ranging from 37.2°C to 40.0°C, with a mean temperature of 37.4°C. Among them, only 7 (1.1%) were detected positive by RDT: two with *P. falciparum* or mixed *P. falciparum/non-P. falciparum* species (40 and 120 parasites/µL) and 5 with *non-P. falciparum* species (2400, 4000, 4000, 12,000 and 16,000 parasites/µL). Ten additional malaria cases were found by using PCR (17/621, 2.7%): 3 with *P. falciparum* (32, 35 and 40 parasites/µL) and 7 with *P. vivax* (30, 32, 35, 40, 48, 53 and 187 parasites/µL). Detailed information including microscopic results is presented in Table 1.

**Table 1:** Characteristics of individuals screened during the FSAT project and prevalence of malaria-infected people detected by RDT among febrile individuals, Pailin, Cambodia, 2010.

Village ID	Village name	Incidence classification from health system (2009)	Population size	Sample collection		Age (years)	Gender
				Number	%	Mean	M/F ratio
							
1	Krachab Krom	High Risk HC	767	701	91.4%	23.9	0.92
2	Phnom Dambang	High Risk HC/VMW	525	375	71.4%	22.6	1.16
3	Oh Tatus	Low Risk	174	140	80.4%	25.1	1.31
4	Oh Preus	High Risk VMW	997	713	71.5%	23.6	1.03
5	Krachab Leu	High Risk HC	529	380	71.8%	23.5	1.05
6	Rothkros Chhes	Low Risk	210	139	66.2%	25.2	1.10
7	Phnom Rang	High Risk HC	428	334	78.0%	24.4	1.10
8	Oh Sour Sdey	High Risk VMW	347	229	65.9%	23.2	0.97
9	Prey Mang Kul	High Risk HC/VMW	459	359	78.2%	24.9	0.99
10	Andong Thmor	High Risk VMW	412	281	68.2%	22.8	1.08
11	Oh Tontram Dey	Low Risk	196	127	64.8%	24.2	0.94
12	Oh Roel	High Risk HC	1022	731	71.5%	24.3	1.04
13	Bor Thmey	High Risk HC	392	282	71.9%	23.3	0.87
14	Oh Treng	High Risk HC/VMW	101	69	68.3%	22.2	1.34
15	Oh Char Kandal	Low Risk	422	306	72.5%	24.5	0.94
16	Pekiri	High Risk VMW	771	530	68.7%	23.0	1.07
17	Bor Tang Sou	High Risk HC	662	445	67.2%	23.2	1.03
18	Andong Py	High Risk VMW	387	236	60.9%	23.9	1.04
19	Phitas Sbov	Low Risk	413	306	74.1%	23.2	1.17
20	Phnom Spung	High Risk HC	323	248	76.8%	22.8	1.12
			**9537**	**6931**	**72.6%**	**23.7**	**1.04**

Risk classification				RDT (if T°>37,2°C)			Microscopy results (parasite/µL)	
A^a^	B	C	D	performed (%)	positive (% )		*P. falciparum*	*P. vivax*
					falciparum	Non-falciparum		
0,0%	0.0%	97.1%	2.9%	104 (14.8%)	0	0	-	-
0.0%	5.9%	88.0%	6.1%	41 (10.9%)	0	1 (2.4%)	-	4000
0,0%	0.6%	97.1%	2.3%	17 (12.1%)	0	0	-	-
0.0%	1.4%	95.3%	3.3%	82 (11.4%)	0	2 (2.4%)	-	16000 & 4000
0,0%	0.0%	96.2%	3.8%	32 (8.4%)	0	0	-	-
0,0%	0.0%	97.6%	2.4%	6 (4.3%)	0	0	-	-
0,0%	0.5%	96.9%	2.6%	36 (10.8%)	0	0	-	-
0.0%	2.6%	90.7%	6.7%	12 (5.2%)	0	1 (8.3%)		12000
0,0%	0.2%	96.1%	3.7%	31 (8.6%)	0	1 (3.2%)		2400
0.0%	3.1%	93.5%	3.4%	42 (14.9%)	0	0	-	-
0,0%	3.0%	92.4%	4.6%	5 (3.9%)	0	0	-	-
0,0%	0.6%	97.5%	1.9%	36 (4.9%)	1 (2.8%)	0	40	
0,0%	1.5%	96.2%	2.3%	7 (2.5%)	1 (14.3%)	0	120	
0.0%	0.0%	93.0%	7.0%	4 (5.8%)	0	0	-	-
0,0%	0.7%	97.4%	1.9%	1 (0.3%)	0	0	-	-
0.0%	1.3%	93.9%	4.8%	56 (10.6%)	0	0	-	-
0,0%	0.6%	95.3%	4.1%	14 (3.1%)	0	0	-	-
0.0%	0.5%	95.4%	4.1%	18 (7.6%)	0	0	-	-
0,0%	5.1%	87.1%	7.8%	50 (16.3%)	0	0	-	-
0,0%	3.1%	93.8%	3.1%	27 (10.9%)	0	0	-	-
**0.0%**	**1.6%**	**94.6%**	**3.8%**	**621 (8.9%)**	**2**	**5**		

a Three persons (0. 03%, 1 in Phnom Dambang and 2 in Oh Preus) were said to work as maids in Malaysia and were initially falsely classified as “A” but have to remain unclassified. However, given that they were most likely to work in an urban area they were considered not be posing a risk to the spread of malaria parasites from Pailin.

Analysis of 6931 dry blood samples by nested-PCR showed 133 (1.9%) positive samples (Table 2): 60 individuals were infected by *P. falciparum* (0.9%, geometric mean of parasite density = 47 parasites/µL, range = 20–1,200 parasites/µL, interquartile range = 32–52 parasites/µL), 72 by *P. vivax* (1.0%, geometric mean of parasite density = 66 parasites/µL, range = 15–1,600 parasites/µL, interquartile range = 32–107 parasites/µL) and one by *P. malariae* (0.01%, parasite density = 45 parasites/µL). The mean time between sample collection and treatment was 8 days (SD±4 days).

**Table 2:** Prevalence of *Plasmodium* carriers (*P. falciparum*, *P. vivax & P. malariae*) detected by PCR among 6931 screened individuals, Pailin, Cambodia, 2010.

Village ID	Village name	Incidence classification from health system 2009	Population size	No. of sample collected	PCR results							
					Parasite carrier		*P. falciparum*		*P. vivax*		*P. malariae*	
					No.	%	No.	%	No.	%	No.	%
1	Krachab Krom	High Risk HC	767	701	9	1.3%	2	0.3%	7	0.9%	0	0.0%
2	Phnom Dambang	High Risk HC/VMW	525	375	13	3.4%	0	0.0%	13	3.4%	0	0.0%
3	Oh Tatus	Low Risk	174	140	7	5.0%	0	0.0%	7	5.0%	0	0.0%
4	Oh Preus	High Risk VMW	997	713	5	0.7%	0	0.0%	5	0.7%	0	0.0%
5	Krachab Leu	High Risk HC	529	380	1	0.3%	0	0.0%	1	0.3%	0	0.0%
6	Rothkros Chhes	Low Risk	210	139	0	0.0%	0	0.0%	0	0.0%	0	0.0%
7	Phnom Rang	High Risk HC	428	334	0	0.0%	0	0.0%	0	0.0%	0	0.0%
8	Oh Sour Sdey	High Risk VMW	347	229	7	3.0%	4	1.7%	3	1.3%	0	0.0%
9	Prey Mang Kul	High Risk HC/VMW	459	359	6	1.7%	0	0.0%	6	1.7%	0	0.0%
10	Andong Thmor	High Risk VMW	412	281	9	3.2%	2	0.7%	7	2.5%	0	0.0%
11	Oh Tontram Dey	Low Risk	196	127	17	13.4%	2	1.6%	15	11.8%	0	0.0%
12	Oh Roel	High Risk HC	1022	731	7	0.9%	3	0.4%	4	0.5%	0	0.0%
13	Bor Thmey	High Risk HC	392	282	9	3.2%	8	2.8%	1	0.3%	0	0.0%
14	Oh Treng	High Risk HC/VMW	101	69	0	0.0%	0	0.0%	0	0.0%	0	0.0%
15	Oh Char Kandal	Low Risk	422	306	4	1.3%	4	1.3%	0	0.0%	0	0.0%
16	Pekiri	High Risk VMW	771	530	37	6.9%	33	6.2%	3	0.5%	1	0.1%
17	Bor Tang Sou	High Risk HC	662	445	2	0.4%	2	0.4%	0	0.0%	0	0.0%
18	Andong Py	High Risk VMW	387	236	0	0.0%	0	0.0%	0	0.0%	0	0.0%
19	Phitas Sbov	Low Risk	413	306	0	0.0%	0	0.0%	0	0.0%	0	0.0%
20	Phnom Spung	High Risk HC	323	248	0	0.0%	0	0.0%	0	0.0%	0	0.0%
			**9537**	**6931**	**133**	**1.9%**	**60**	**0.86%**	**72**	**1.04%**	**1**	**0.01%**

### Malaria epidemiology

#### Malaria positive cases

**Figure 2:**
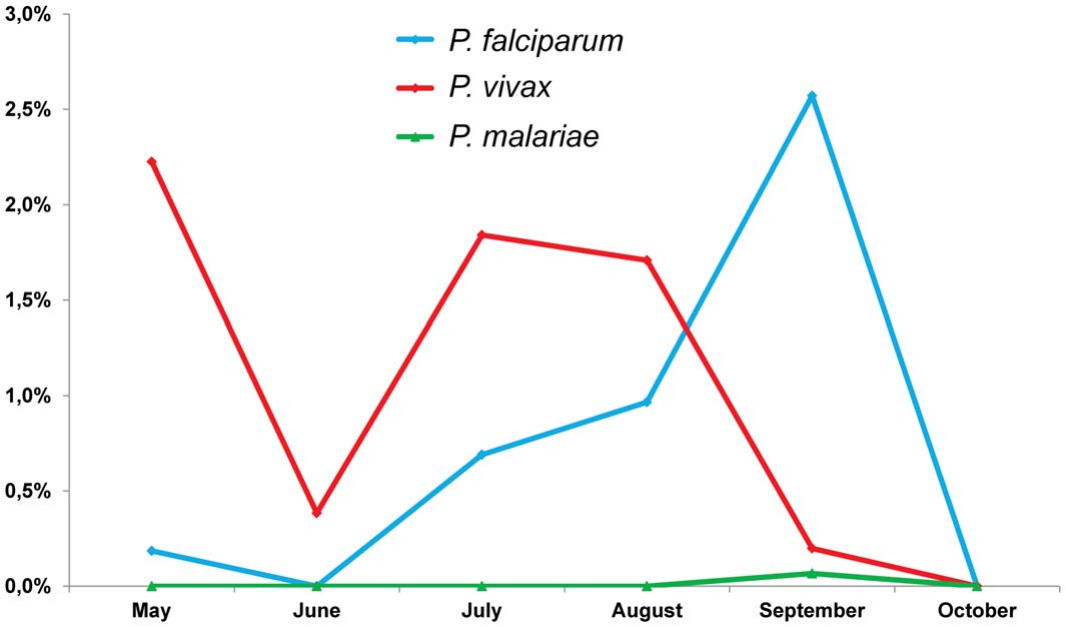
Temporal evolution of the proportion of malaria parasites carriers, Pailin, Cambodia, 2010.

Six out of the 20 villages screened were free of malaria parasites: Rothkros Chhes, Phnom Rang, Oh Treng, Andong Py, Phitas Sbov and Phnom Spung. Temporal evolution of the proportion of malaria parasites carriers showed significant difference according to the month of the collection for the most prevalent species, *P. falciparum* (*P* = 0.0001) and *P. vivax* (*P* = 0.0003) (Figure 2). All malaria positive cases had declared having stayed or were likely to stay within Pailin or local transmission areas within Cambodia.

#### *P. falciparum* cases

Sixty *P. falciparum* infections were found in 9 villages (11 out of the 20 villages were *P. falciparum* cases free). The prevalence of *P. falciparum* infections was not significantly different between adults (0.94%) and children under the age of 15 (0.74%) (*P* = 0.36) and between males (1.02%) and females (0.72%) (*P* = 0.18).

**Figure 3:**
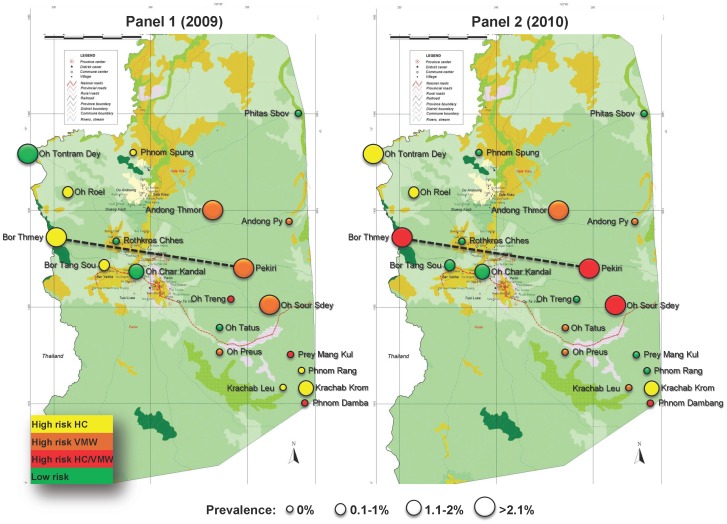
Spatial distribution of *P. falciparum* prevalence according to malaria incidence reported through HC and VMW in 2009 (Panel 1) and in 2010 (Panel 2), Pailin, Cambodia, FSAT 2010.

According to the classification of villages, the mean prevalence of asymptomatic parasite carriers was not significantly different between “high risk” villages and “low risk” villages based on HC 2009 data (0.38% *vs.* 0.59%, *P* = 0.41). In contrast, there was a statistically significant correlation between the mean prevalence of parasite carriers of “high risk” and “low risk” villages identified by VMW in 2009 (1.41% *vs.* 0.59%, *P* = 0.04). While the FSAT investigation was being conducted from May to end of October 2010 detecting almost exclusively asymptomatic parasite carriers, the disease surveillance system in Pailin continued to detect and parasitologically confirm the incidence of symptomatic carriers. At the end of the year a re-classification of high and low risk villages became possible based on the newly available incidence data of 2010 using the same selection criteria as for 2009 incidence reports. Even though the reported incidence of *P. falciparum* had fallen by 73.4% (from 941 to 250) in 2010, 11 out of the initial 15 high risk villages remained among the ten with highest case reports in the two HC and VMW disease surveillance systems (Malaria Consortium, unpublished data). The analysis of the FSAT results showed that the correlation between the prevalence of asymptomatic carriers and disease incidence reports (both surveillance systems) of the same year was highly significant. “High risk HC 2010” villages had on average six times more asymptomatic carriers than “low risk HC 2010” villages (1.57% vs. 0.24%, *P*<10^−4^). A very similar result was observed when comparing “high risk VMW 2010” villages with “low risk VMW 2010” villages (1.48% *vs.*0.24%, P<10^−4^) (Table S2). Based on 2010 cases report, the prevalence was significantly higher in “high risk HC/VMW 2010” villages compared to “low risk HC/VMW 2010” villages (1.10% *vs.* 0.27%, *P* = 0.005). *P. falciparum* prevalence was not significantly different between adults and children (0.94% vs. 0.74%, *P* = 0.36) and between males and females (1.02% vs. 0.72%, *P* = 0. 18). The spatial distribution of *P. falciparum* prevalence according to malaria incidence reported through HC and VMW in 2009 (Panel 1) and in 2010 (Panel 2) is shown in Figure 3.

#### *P. falciparum* genetic diversity and gene flow

Eight-locus genotyping of *P. falciparum* isolates displayed 17 different haplotypes. Twelve haplotypes (70%) were found solely in several villages: Pekiri (N°3, 4, 9 & 11), Bor Thmey (N°2, 12, 13, 14 & 17), Andong Thmor (N°8 & 16) and Oh Chrakandal (N°15) while the others were shared between villages: N°1 (Oh Sour Sdey & Pekiri), N°5 (Bor Tang Sou & Pekiri), N°6 (Bor Thmey, Krachab Krom, Oh Chrakandal, Oh Roel & Pekiri), N°7 (Bor Thmey, Krachab Krom, Oh Soursdey & Pekiri), N°10 (Bor Tangsou, Bor Thmey, Oh Roel, Oh Soursdey, Oh Tantramdey & Pekiri). Genetic diversity, assessed by Nei's unbiased expected heterozygosity (He) ranged from 0.06 (Oh Roel) to 0.33 (Bor Thmey) and was significantly higher in Bor Thmey compared to Pekiri (0.33±0. 9 *vs.* 0.19±0.12, *P* = 0.01, Table 3). The degree of genetic differentiation within *P. falciparum* populations, estimated by F_st_ values, indicated divergence between villages, except for Bor Thmey and Pekiri (F_st_ = 0.00139, *P*>0. 05), highlighting a parasite flow between these two villages.

**Table 3:** Genetic diversity at village level expressed as mean expected Heterozygosity (He) using eights SNPs in *P. falciparum* collected in Pailin, Cambodia, 2010.

Village	No. of *P. falciparum*	No. of haplotypes	Mean *He* of 8 loci (±SD)
Andong Thmor	2	2	0. 16±0. 31
Bor Tang Sou	2	2	0. 16±0. 31
Bor Thmey	8	8	0. 33±0. 19
Krachab Krom	2	2	0. 08±0. 23
Oh Char Kandal	4	2	0. 14±0. 26
Oh Roel	3	2	0. 06±0. 18
Oh Sour Sdey	4	3	0. 23±0. 25
Oh Tontram Dey	2	1	-
Pekiri	33	9	0. 19±0. 12

#### *P. vivax* cases

**Figure 4:**
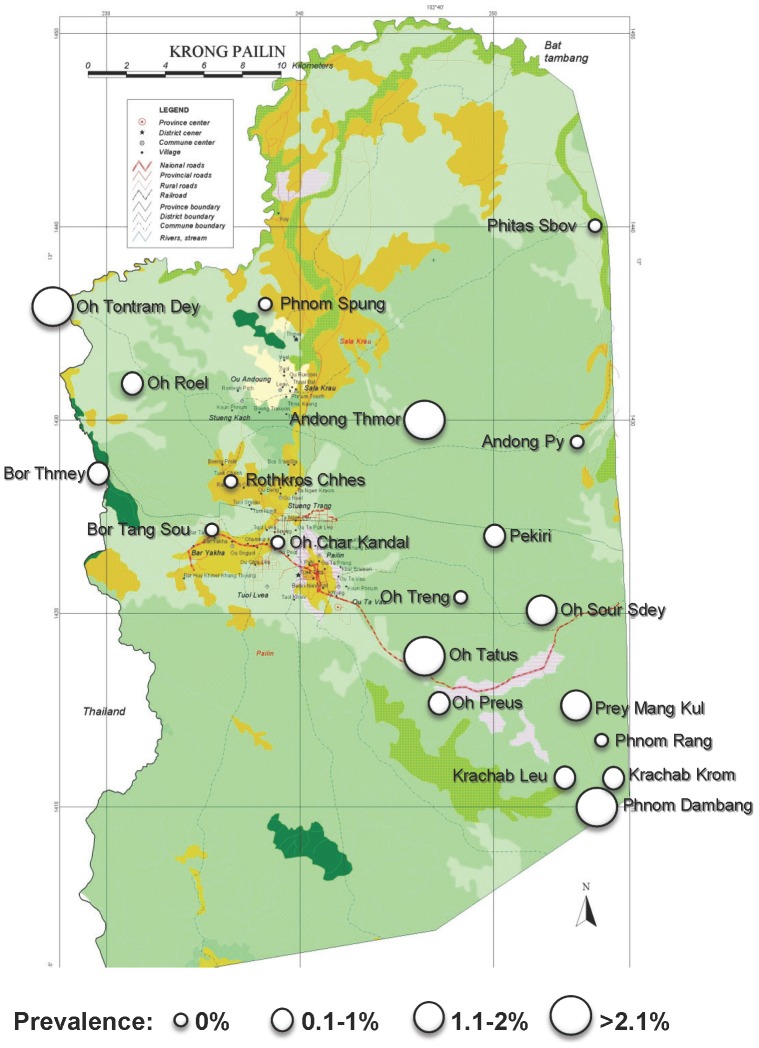
Spatial distribution of *P. vivax* prevalence, Pailin, Cambodia, 2010.

Seventy two *P. vivax* infections were found in 12 villages (8 out of the 20 villages screened had no *P. vivax* cases). *P. vivax* infections were significantly more prevalent in adults (1.24% *vs.* 0.74%, *P* = 0.046), but no significant difference was found between males and females (1.13% *vs.* 0.80%, *P* = 0.15). Spatial distribution of *P. vivax* prevalence is shown in Figure 4 (Panel B).

#### G6PD deficiency screening

Screening of G6PD deficiency by using blood spots among the malaria infected cases gave results which were considered skewed towards artificially low G6PD enzyme activity levels due to methodical problems, as explained in the discussion section. The global prevalence of G6PD deficient people among parasites carriers was estimated to 63% (18% with 10%–30% of enzyme activity and 45% <10% of enzyme activity).

#### Polymorphism at codon 268 in cytb gene

Among the 60 *P. falciparum* cases, all parasites were wild-type (Y268).

#### Treatment outcomes of *P. falciparum* cases

Five different antimalarial regimens were given to 57 of 60 *P. falciparum* cases: AP (n = 25), AP+PQ (n = 25), CQ (n = 1, wrongly given), QN (n = 1, pregnant woman) and DHA-PIP (n = 5, at the beginning of the study when AP was not available). Three individuals were lost (had left their villages to Preah Vihear, n = 1 or Kampot provinces, n = 2). The 28-day follow-up data were available for 51 patients. One patient treated with DHA-PIP failed and was classified as treatment failure following genotyping. One patient treated with AP developed vivax malaria at day 28.

#### Treatment outcomes of *P. vivax* cases

For *P. vivax* cases, 59 patients received CQ alone and only 11 patients received CQ with PQ due to the many false positive G6PD tests. Two individuals were lost (had left their villages to Prey Veng, n = 1 or Battambang provinces, n = 1). The 28-day follow-up data were available for 64 patients. Both CQ and CQ+PQ were 100% effective in clearing *P. vivax* infections.

## Discussion

The emergence of artemisinin resistance in western Cambodia demanded urgent action to contain its spread and eliminate malaria in identified foci [21]. To contain this threat, a number of strategic priorities were identified and implemented rapidly, such as the mass deployment of LLINs, the banning artemisinin monotherapies and the strengthening of public sector provision of free parasitological diagnosis and treatment through VMWs and HCs. However, there was a paucity of epidemiological information on which to base the planning of additional interventions and a lack of operational experience in Cambodia and elsewhere in implementing potential new tools for elimination. At this stage PCR based diagnosis was limited to epidemiologically explore the reservoir of parasites in asymptomatic carriers.

The most encouraging epidemiological insight gained from FSAT was to that seems extremely low. Out of the 6,931 persons screened and among the 2,606 family relatives of the screened who were absent at the time of screening, none had any travel history to or from Myanmar. Although there is long history of presence of Burmese gem miners in Pailin [22]–[24], two widely corroborated phenomena may explain the complete absence, since several years now, of any Burmese in Pailin: (i) the former abundance of rubies and others gems has now been depleted and (ii) alternative attractive economic opportunities do not exist in Pailin for foreigners, due to lower salary levels when compared to similar activities on the Thailand side of the border.

There had also been concerns that artemisinin resistance might spread indirectly from Palin to Myanmar via temporary or permanent residents of Pailin when migrating to areas of local transmission on the Thailand side of the border, to work in orchards, rubber or plantations or in the fishing industry at the coast in which many Burmese are working [25]–[27]. Here again, the results were encouraging as only as 139 (1.47%) of the 9537 persons registered through the survey qualified to be included into the “risk group B” for AR spread as “likely to go to malaria transmission areas in Thailand”. All malaria positive cases reported to have stayed in Pailin or local transmission areas in Cambodia in the preceding year. Only 8/6931 persons tested (0.12%) had a travel history to Thailand, all of them being PCR negative. All others did not have any travel history that could pose a risk to containment of *P. falciparum* spread outside of Pailin. However, temporary migrations of seasonal workers from Cambodia to Thailand seem to be likely responsible for the parasite gene flow that we have observed between villages along the Cambodian-Thailand border (i.e. Bor Thmey) and villages inside Pailin province (i.e. Pekiri).

Until the launch of the FSAT project, there were concerns that the existing health information system (HIS), and in particularly the HC incidence data would not accurately identify foci of infection because it is known that patients in Cambodia seek treatment in the private sector, especially if they are far away from public health facilities [28]–[30]. In addition, the villages with higher burdens of symptomatic malaria – (indicative of relatively non-immune populations) might be different from those villages in which the inhabitants were more immune and had higher levels of asymptomatic carriage. These villages might therefore be harboring a large parasite reservoir. However our results showed a good correlation between the high burden villages as identified by the HIS data on incidence of symptomatic cases of 2010. The passive case detection incidence data recorded by the VMWs was already significantly identifying the villages with higher burdens of asymptomatic carriage in 2009 as well as in 2010.

The VMWs are clearly a critical component of the containment strategy. As shown here, not only are they instrumental in reducing population parasite burden by providing easy access to good quality diagnosis and treatment, but they are a critical component of the surveillance system [31]. Currently, they only operate in the 43 out of 109 villages in Pailin, which are located more than 5 km away from the nearest health center. Given their important role they can play in containment, expansion of the scheme to include all villages in Pailin should be considered [32].

FSAT proved achievable logistically, the complex protocol of screening, treatment and follow up of over 6'000 individuals was sustained over six months. In addition, FSAT has generated scientific results on patterns of infection and quantified the risks of potential drug resistant parasites spread outside of Pailin. This operational success under difficult field conditions suggests that FSAT might be a feasible method for cross-sectional screening with PCR and directly observed treatment in other settings. A high level of case identification and treatment was achieved despite there being a mean lapse of 8 days between the day of screening and the case being located by the follow-up team. Out of the *P. falciparum* cases that were detected by PCR, 95% could be traced for follow-up. Of those who could remain in the study all adhered to the directly observed three days of treatment. Of these cases, 96% were followed up until day 28. *P. falciparum* parasites circulating in Pailin province were found to be sensitive to atovaquone (none of them being mutant-type at codon 268) and asexual parasites totally sensitive to AP. Only one case of treatment failure was observed from an individual treated with DHA-PIP. This observation was not surprising due to high level of recrudescence (∼25%) recently found in this area (Leang et *al.*, submitted 2012).

A number of important lessons were learnt which may help to inform the future programme implementation. The main challenge was the lack of accurate information on the size and location of the population because of the amount of population movement in the area. As noted elsewhere, much of this movement is dictated by the seasonal agricultural calendar [26]. The estimates in population size therefore varied considerably making it difficult to accurately calculate coverage rates. Population estimates based on a previous census data were on average 43.7% higher (range: 18.5–66.7%) than the estimates made by the FSAT team with the help of village leaders and based on the population estimates during FSAT, the mean coverage rate was 72.1% (range: 61.0%–91.4%). Transporting blood samples from the field for analysis in the laboratory in Phnom Penh also proved problematic, not only because of the resulting delay between screening and treatment of positive cases, but also the accuracy of some of the results, particularly the G6PD results. The high levels of G6PD deficiency reported here compared to the likely true prevalence of around 10%, [33] were thought to be due partly to the high threshold (<30% of the normal enzyme activity) chosen to declare a person as deficient. In addition, a deterioration of the samples during transportation to Phnom Penh, a day's drive away from the villages was very likely. For this reason a mobile laboratory has since been purchased to improve the speed and the reliability of FSAT. In Cambodia, PCR can now be carried out in the field by placing the mobile laboratory within the target villages reducing the interval time between sample collection and treatment of positive cases to 24 hours. In addition, the detection of mutation at codon 268 in cytochrome *b* gene and G6PD deficiency screening can now be similarly carried out within the same time and expected increases in reliability. Moreover, the blood sample analysis within the village is likely to render this practice more acceptable and interesting for the population, increasing the coverage rates.

Given the impossibility of carrying out MSAT at the scale originally envisaged, mainly for reasons of lack of human resources in Cambodia, a strong case has been made in favour of Mass Drug Administration (MDA) throughout Pailin as a radical measure to rapidly eliminate *P. falciparum* from the province. The arguments in favour of MDA include the slowness of FSAT (screening speed of one village per week) and the possibility that there would be asymptomatic carriers under the low parasitaemia threshold of PCR of 0.1 parasites/µl who might escape detection. However, the main difficulty encountered during FSAT (i. e. that of high population movements into, within and out of Pailin) would also be encountered by MDA. But, in contrast to FSAT and MSAT, MDA would also make any epidemiological analysis impossible and effectively render the programme blind as to the location of last remaining foci of transmission. Finally, the rapid geographical widening of drug pressure caused by MDA, followed by a longer period of mass exposure of sub-lethal plasma concentrations of the drug used, present simultaneously in large sections of the population, could trigger the very selection and emergence of multi-drug resistant parasite populations that many so rightly fear. This would be particularly true for AP if it was to be used for MDA, as only one single mutation in the parasite's genome could render this drug ineffective as have successively become all its predecessors throughout Pailin [34].

In conclusion, FSAT may be a useful additional tool in the containment of AR parasites, among the other strategies implemented, albeit its shortcomings in terms of slowness and coverage in three important ways: (i) by providing information on travel and migrations patterns, (ii) by identifying clusters of asymptomatic carriers and (iii) by reducing the overall parasite biomass through the identification and treatment of infections that would otherwise have been undetected. However, recent a report demonstrating the emergence of AR malaria along the Myanmar-Thailand border emphasize the need to expand containment efforts and to further improve surveillance strategies [35]. These might include PCR-based FSAT as new epidemiological tool to help direct containment efforts or malaria elimination in other contexts [36], [37]

## Supporting Information

Table S1 Sequences of primers used for *P. falciparum* bar-coding, Pailin, Cambodia, 2010.
(DOCX)

Table S2 Clustering of asymptomatic carriers based on HC and VMW data in 2009 and 2010, Pailin, Cambodia
(DOCX)
