# Supplementary material for: Focused Screening and Treatment (FSAT): A PCR-Based Strategy to Detect Malaria Parasite Carriers and Contain Drug Resistant P. falciparum, Pailin, Cambodia
Source: PLoS One. 2012 Oct 1;7(10):e45797. doi: 10.1371/journal.pone.0045797 (PMC3462177; doi:10.1371/journal.pone.0045797)
Supplement: Table S1 — Sequences of primers used for P. falciparum bar-coding, Pailin, Cambodia, 2010. (DOCX) [file pone.0045797.s001.docx]

**Table S1.** Sequences of primers used for *P. falciparum* bar-coding, Pailin, Cambodia, 2010.

| **Assays** | **Outer PCR primers (5'-3')** | **Tm** | **Inner PCR primers (5'-3')** | **Tm** | **SNP** |
| --- | --- | --- | --- | --- | --- |
| 3 | TGGAAATACACAATTCAATG | 55°C | TTCCAAAACTATGTTTGCTGCT | 58°C | T/C |
|  | CGAATGTTTTTCCATATTTT |  | TGCAGTGGTACTTGTTGCTACC |  |  |
| 4 | CCAACCAACGAACACAAATAC | 55°C | AGGAAAATGCTCCGGTAACT | 58°C | T/C |
|  | TGGTTGACTGTTATTGGGGTA |  | GGTTCATATTATTTGGTGACTCG |  |  |
| 7 | TGAATGTAATATAAATCAGGTTG | 53°C | CTGAAAAATCGGATGAATGG | 58°C | A/G |
|  | GGCTGGAATAGATAAAATCA |  | GGCTAGCTCAGCTTCCAAT |  |  |
| 8 | CGAATTTAAGTACCTTAGGAAA | 53°C | TCACAACGTCCATATGTTGAA | 58°C | A/G |
|  | TCATAAAGTTTTTATTGTCTTCA |  | TCATTATCACCTACTTTCTGTACCA |  |  |
| 9 | GAGGATGTATACCATTAGCTG | 52°C | GATGAGTTAGCAACGAAACCA | 58°C | T/A |
|  | ATCATTCATATGTGGAAACA |  | AACGTAAACCAGGAGTAAGACG |  |  |
| 19 | TCACAAACAAATAACAATGAA | 52°C | AAAAGCAATTCCACAAGAACC | 55°C | A/C |
|  | ACATGTTTTGGACCATCTAC |  | CTGGTGTTTCCTTTTTATTTGG |  |  |
| 20 | AATATATCTGTATTTGCTAACATGA | 52°C | TGTGTTTTATTTTTAGTGTGAGCTTT | 57°C | C/T |
|  | TGTAACAAGGAATGACAAAA |  | AGAGGATATCCAATAGGGTGCT |  |  |
| 24 | CGATTTAATTACTGTTTTGAGA | 52°C | AACAAATCATCAATTAAGTCATCC | 55°C | G/T |
|  | TTGGTTTACAATTAGTTCTAGC |  | TGAGGAATAGGTTCATATGCTG |  |  |
