# Supplementary material for: Focused Screening and Treatment (FSAT): A PCR-Based Strategy to Detect Malaria Parasite Carriers and Contain Drug Resistant P. falciparum, Pailin, Cambodia
Source: PLoS One. 2012 Oct 1;7(10):e45797. doi: 10.1371/journal.pone.0045797 (PMC3462177; doi:10.1371/journal.pone.0045797)
Supplement: Table S2 — Clustering of asymptomatic carriers based on HC and VMW data in 2009 and 2010, Pailin, Cambodia (DOCX) [file pone.0045797.s002.docx]

**Table S2.** Clustering of asymptomatic carriers based on HC and VMW data in 2009 and 2010, Pailin, Cambodia

| Villages classification based on | | FSAT 2010 results | | *P-*value^a^ |
| --- | --- | --- | --- | --- |
|  |  | *Pf* positive (%) | *Pf* negative |  |
| HC 2009 data | High Risk* | 15 (0.38) | 3909 | 0.41 |
|  | Low Risk | 6 (0.59) | 1012 |  |
| HC 2010 data | High Risk* | 52 (1.57) | 3261 | < 10^-4^ |
|  | Low Risk | 4 (0.24) | 1649 |  |
| VMW 2009 data | High Risk** | 39 (1.40) | 2753 | 0.04 |
|  | Low Risk | 6 (0.59) | 1012 |  |
| VMW 2010 data | High Risk** | 47 (1.48) | 3119 | < 10^-4^ |
|  | Low Risk | 4 (0.24) | 1649 |  |

* Village ranked among the top ten villages among Pailin's 109 villages reporting incidence of microscopy confirmed *Pf* malaria cases at the health centres of Pailin and in districts neighbouring Pailin; ** Village ranked among the top ten villages among Pailin's 43 villages situated at more than 5 km from health centres and therefore supported by Village Malaria Health workers reporting the incidence of RDT confirmed *Pf* malaria cases within their village ; ^a^ P-value - Fisher exact test
